# Multimodal Approach to Vertebral Body Tethering With Erector Spinae Plane Blocks and Cryoablation

**DOI:** 10.7759/cureus.31260

**Published:** 2022-11-08

**Authors:** Adam P Chao

**Affiliations:** 1 Pediatric Anesthesiology, Shriners Children's, Sacramento, USA

**Keywords:** pediatric regional anesthesia, multimodal analgesia, pediatric scoliosis, cryoablation, vertebral body tethering, erector spinae plane block

## Abstract

Multimodal analgesia that combines around-the-clock medications and regional techniques can be especially effective for postoperative pain control. We describe a pediatric patient who underwent vertebral body tethering via an open thoracolumbar approach to treat juvenile idiopathic scoliosis. Erector spinae plane blocks (ESPBs), cryoablation to the intercostal nerves, and multimodal medications helped control our patient’s pain well enough for her to be discharged home on postoperative day 2. To the best of our knowledge, this is the first report of this combination of regional techniques used for vertebral body tethering (VBT).

## Introduction

Postoperative pain is caused by a combination of inflammatory, nociceptive, and neuropathic pathways [1]. Modulation of these different pathways via regional and pharmacologic techniques can decrease postoperative pain scores and shorten hospital stays [2]. In this case report, we describe an analgesic approach combining the erector spinae plane block (ESPB) with liposomal bupivacaine, cryoablation, and around-the-clock medications. We chose to use the ESPB in our analgesic plan as it has been shown to decrease postoperative pain scores and opioid consumption in a variety of spinal surgeries [3] and could provide early analgesia before the cryoablation performed intraoperatively to the intercostal nerves was expected to take full effect. Vertebral body tethering (VBT) is a novel growth modulation technique used to treat adolescent idiopathic scoliosis. The surgical technique involves placing screws on the convex side of a scoliotic curve and tethering them together with a cord, inhibiting growth on the convex side while permitting the concave side to grow and lengthen [4]. Over time, this progressively reduces the degree of scoliosis.

## Case presentation

Our patient was a 12-year-old, 41 kg American Society of Anesthesiologists (ASA) physical status I female with juvenile idiopathic scoliosis who had a Lenke type V curve with lumbar and thoracic curves of 52 and 32 degrees, respectively. She had no other pertinent past medical or surgical history. Despite bracing, her curve had progressed over the years, and she presented to our hospital for VBT of T11-L4.

**Figure 1:**
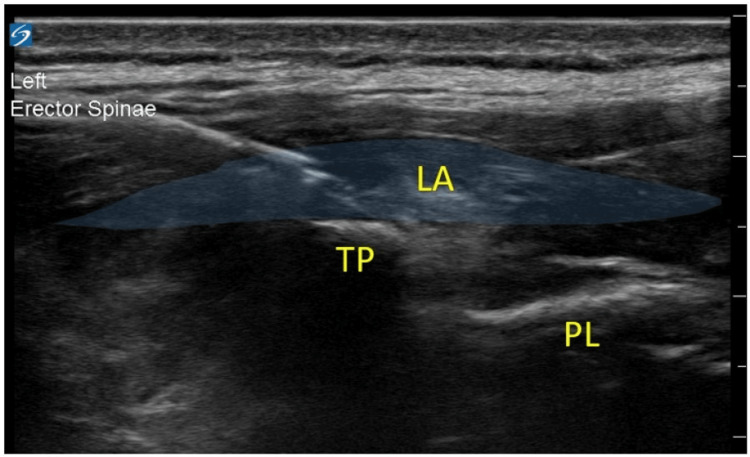
Ultrasound-guided thoracic ESPB at T11 - injection of local anesthetic over transverse process. LA, local anesthetic; TP, transverse process; PL, pleura.

After premedication with intravenous (IV) midazolam 2 mg, we brought her to the operating room, placed standard ASA monitors, and induced general anesthesia with standard medications, after which we performed oral endotracheal intubation. A radial arterial line was placed. We then administered ketamine 1 mg/kg IV, methadone 0.2 mg/kg IV, and dexamethasone 0.2 mg/kg IV as part of our multimodal analgesic regimen. The ketamine was continued as an infusion at 7 mcg/kg/min IV. To perform the ESPBs, we used a linear high-frequency ultrasound probe to identify the vertebral spinous processes, then scanned laterally towards the side of the incision and identified the transverse processes and overlying erector spinae muscles. We advanced a 22-gauge, 90-mm needle until contact with the transverse process of the T11 vertebra was achieved, and then injected several milliliters of normal saline to ensure the needle tip was in the correct fascial plane. Then, we injected a 20-mL mixture of 6 mL of 1.33% liposomal bupivacaine and 14 mL of 0.25% bupivacaine (Figure 1). The second ESPB was performed in a similar fashion over the L2 transverse process with the same volume and mixture of local anesthetic. Of note, the T11 and L2 levels were chosen such that the ESPBs performed at these points would approximately cover the planned incision as marked by the surgeon.

**Figure 2:**
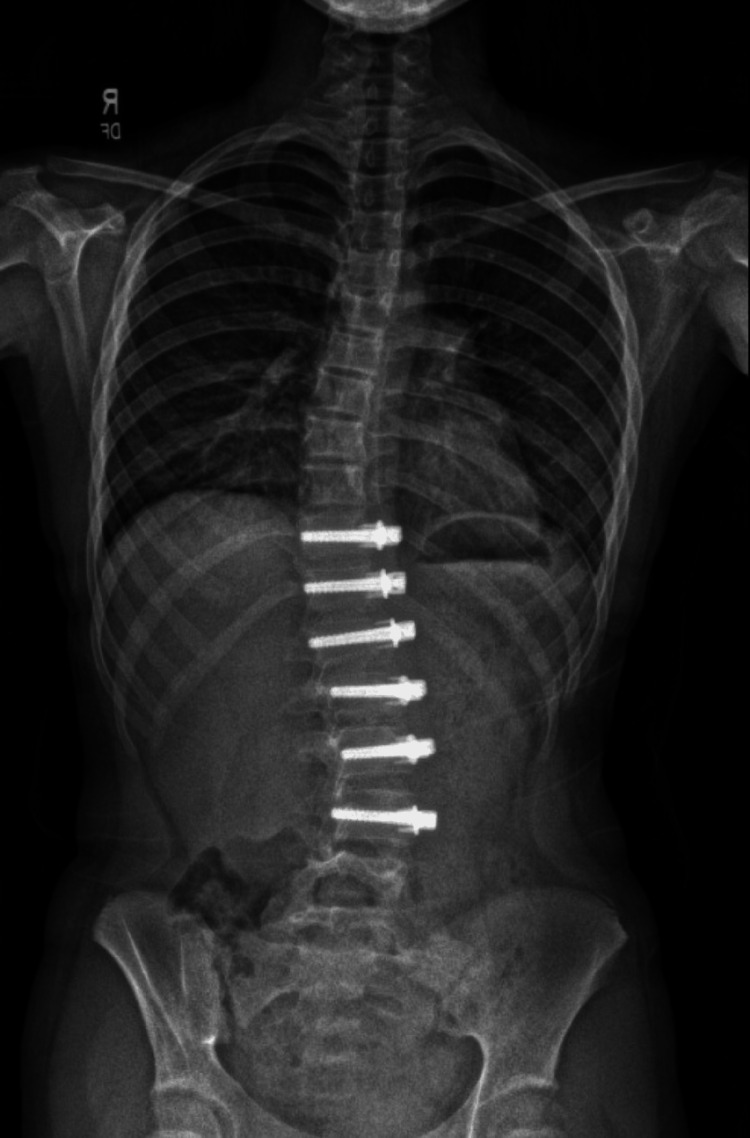
Post-operative X-ray with instrumentation T11-L4.

For the surgical approach, the patient was placed in the right lateral decubitus position, and a left thoracoabdominal incision was made spanning from the level of the iliac crest to the angle of the scapula. A thoracotomy was made through the ninth rib interspace over the top of the tenth rib, the tenth rib was cut with a rib cutter, and the neurovascular bundle was ligated. The diaphragm was taken down, and the retroperitoneal space was entered. Of note, single-lung ventilation was not required as the majority of the surgery was performed outside the thoracic cavity. Six bicortical screws were inserted through the lateral walls of the vertebral bodies from T11 to L4 and then tethered via a cord (Figure 2). Before closing the incision, the surgeon performed cryoablation on the directly visualized intercostal nerves. A single chest tube was placed. The total anesthetic time was 315 minutes, and the estimated blood loss was 150 mL. For her analgesic medications postoperatively, she was given gabapentin 5 mg/kg per os three times a day, acetaminophen 10 mg/kg per os q6h, and ketorolac 0.5 mg/kg IV q6h, but opioids and benzodiazepines were only ordered pro re nata. The intraoperative ketamine infusion was not continued.

**Figure 3:**
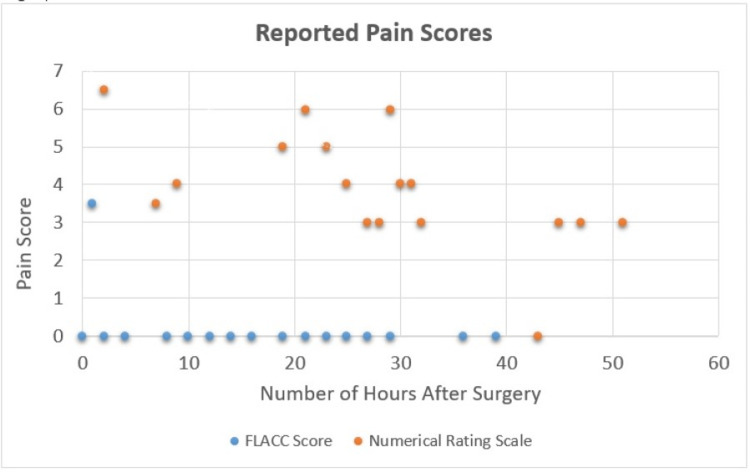
Reported pain scores during hospital admission. FLACC: Faces, Legs, Activity, Cry, Consolability Scale.

Our patient’s faces, legs, activity, crying, consolability (FLACC), and pain numerical rating scale values in the hospital are illustrated in Figure 3. Her FLACC scores were zero throughout her admission except for one value in the post-anesthesia care unit, and her pain numerical rating scale values ranged from three to six (on a scale of 0-10). Of note, she received only one dose of an opioid during her entire hospital admission (hydromorphone 0.5 mg IV on postoperative day 0) and one dose of a benzodiazepine (diazepam 2 mg per os on postoperative day 1). This minimal use of opioids and muscle relaxants is consistent with her reports that she had some minor soreness that was tolerable. On postoperative day 2, she was uneventfully discharged after meeting all surgical discharge criteria. In comparison, a multicenter review from 2020 of 57 consecutive patients undergoing anterior VBT showed a mean length of stay of 4.7 days (SD 1.4; 3 to 9) [5].

At the telephone follow-up on postoperative day 5, her pain was still well-controlled, and she had used a total of three doses of oxycodone (5 mg per os) and three doses of diazepam (2 mg per os). She did not experience any untoward side effects of nausea, respiratory depression, or dizziness, but she did endorse having some constipation. At her four-week clinic follow-up, she had no pain, and she had stopped taking her prescribed ibuprofen and acetaminophen. At her three-month and six-month follow-up appointments, she also had no pain and was not taking any medications.

## Discussion

The ESPB is a regional anesthetic technique first described for successful use in 2016 to manage thoracic neuropathic pain in a patient with metastatic disease to the ribs [6]. While the exact mechanism of action of the ESPB is not completely understood, it is likely due to a combination of effects such as local anesthetic spread to the ventral rami, dorsal rami, and less commonly the epidural space [7]. ESPBs performed with liposomal bupivacaine can be expected to cover somatic as well as visceral pain for 48-72 hours [8]. We chose to perform ESPBs with liposomal bupivacaine in our analgesic plan as it can provide immediate and early analgesia before the effects of cryoablation on the intercostal nerves were expected to take full effect. While an epidural is also a regional technique that may work for VBT, the ESPB has the advantage of avoiding risks such as epidural hematoma formation, inadvertent dural puncture, urinary retention, and lower extremity weakness leading to delayed ambulation.

To date, there is no current literature describing the combination of the ESPB, cryoablation, and multimodal medications as part of an analgesic plan for vertebral body tethering. A case series by Lee and Kydes [9] examined continuous bilateral erector spinae plane catheters compared with intercostal nerve blocks performed with liposomal bupivacaine for vertebral body tethering, with both techniques leading to satisfactory pain relief. However, our analgesic plan is unique in that it could provide long-term analgesia by incorporating cryoablation and also included medications with pain-modulating properties such as methadone, ketamine, and gabapentin. 

Cryoablation involves a technique where high-pressure carbon dioxide or nitrous oxide is applied directly to tissue overlying a nerve, rapidly cooling it to −50 to −70 °C and causing axonotmesis and Wallerian degeneration. This leads to a temporary disruption of pain transmission. The outer neuronal structure remains intact, and axonal regeneration is often seen in four to six weeks [10]. In the past, cryoablation to the intercostal nerves has been used to treat post-thoracotomy pain and for post-operative analgesia after Nuss bar insertions [11]. The expected duration of analgesia to the chest wall is two to twelve months, but it usually does not take full effect until 24 hours after the procedure [12]. Thus, it cannot be used as a sole technique for pain control. The risks of the procedure are very low because it is performed under direct or video visualization and because the cellular damage is not permanent.

Medications utilized in our opioid-sparing plan include methadone, ketamine, and around-the-clock gabapentin, acetaminophen, and ketorolac. These medications have different mechanisms of action and have a synergistic effect when administered together. We will briefly review some of their important characteristics.

Methadone is an opioid with a long elimination half-life of 24-36 hours [13]. The duration of action of methadone is variable depending on the dose [14], and we used a moderate dose of 0.2 mg/kg, which we expected to have a therapeutic effect of one to two days. In addition to being a mu agonist, methadone is also an N-methyl-d-aspartate (NMDA) receptor antagonist and has been shown to decrease neuropathic pain [15]. A single dose of methadone, compared to multiple repeated doses of shorter-acting opioids, also yields a more predictable blood concentration level of opioids and is less likely to produce inadvertent respiratory depression.

Ketamine, like methadone, is also an NMDA receptor antagonist and is expected to decrease the risk of postoperative chronic and neuropathic pain with its antihyperalgesic and antiallodynic properties [16]. Gabapentin is an anticonvulsant, a ligand of the α2δ calcium channel subunit, and has been shown to decrease perioperative central sensitization and post-surgical neuropathic pain [17]. These medications were an important part of our plan as thoracotomies are commonly associated with post-operative neuropathic pain, affecting up to 57% of patients at three months and 47% of patients at six months [18].

While acetaminophen and ketorolac are two common medications used to treat mild or moderate pain, they are a frequent component of enhanced recovery after surgery protocols because of their opioid-sparing qualities and have been shown to promote earlier ambulation after colorectal surgery [19]. Acetaminophen’s mechanism of action is not completely understood, but it is likely related to its ability to inhibit the cyclooxygenase pathway and inhibit the synthesis of prostaglandins in the central nervous system, leading to analgesic and antipyretic effects [20]. Ketorolac is an intravenous nonsteroidal anti-inflammatory drug that also works via the inhibition of cyclooxygenase, preventing the enzymatic conversion of arachidonic acid into thromboxanes, prostaglandins, and prostacyclins. A decrease in these eicosanoids leads to the medication’s analgesic, antipyretic, and anti-inflammatory effects [21]. Ketorolac, as with all nonsteroidal anti-inflammatory medications, may increase the risk of peptic ulcers and bleeding and should be used with caution in asthmatics and those with renal disease.

## Conclusions

An analgesic plan, when at all possible, should involve regional techniques and pharmacology that target different pathways involved in pain transmission. Our analgesic plan for vertebral body tethering greatly reduced the post-operative opioid needs of our patient, and it also facilitated her discharge from the hospital on postoperative day 2 after major surgery. We hope that this case report inspires similar multimodal analgesic plans for vertebral body tethering, although further research is needed to determine the best approach to pain management in these procedures.

## References

[1] Mathiesen O, Dahl B, Thomsen BA, Kitter B, Sonne N, Dahl JB, Kehlet H (2013). A comprehensive multimodal pain treatment reduces opioid consumption after multilevel spine surgery. Eur Spine J.

[2] Joshi GP, Kehlet H (2019). Enhanced recovery pathways: looking into the future. Anesth Analg.

[3] Liang X, Zhou W, Fan Y (2021). Erector spinae plane block for spinal surgery: a systematic review and meta-analysis. Korean J Pain.

[4] Stokes IA, Spence H, Aronsson DD, Kilmer N (1996). Mechanical modulation of vertebral body growth. Implications for scoliosis progression. Spine (Phila Pa 1976).

[5] Miyanji F, Pawelek J, Nasto LA, Rushton P, Simmonds A, Parent S (2020). Safety and efficacy of anterior vertebral body tethering in the treatment of idiopathic scoliosis. Bone Joint J.

[6] Forero M, Adhikary SD, Lopez H, Tsui C, Chin KJ (2016). The erector spinae plane block: a novel analgesic technique in thoracic neuropathic pain. Reg Anesth Pain Med.

[7] Chin KJ, El-Boghdadly K (2021). Mechanisms of action of the erector spinae plane (ESP) block: a narrative review. Can J Anaesth.

[8] Boezaart AP, Smith CR, Chembrovich S (2021). Visceral versus somatic pain: an educational review of anatomy and clinical implications. Reg Anesth Pain Med.

[9] Lee NS, Kydes A (2021). Regional anesthetic approaches for postoperative analgesia following vertebral body tethering: a case series. A A Pract.

[10] Moorjani N, Zhao F, Tian Y, Liang C, Kaluba J, Maiwand MO (2001). Effects of cryoanalgesia on post-thoracotomy pain and on the structure of intercostal nerves: a human prospective randomized trial and a histological study. Eur J Cardiothorac Surg.

[11] Graves CE, Moyer J, Zobel MJ, Mora R, Smith D, O'Day M, Padilla BE (2019). Intraoperative intercostal nerve cryoablation during the Nuss procedure reduces length of stay and opioid requirement: a randomized clinical trial. J Pediatr Surg.

[12] Sujka J, Benedict LA, Fraser JD, Aguayo P, Millspaugh DL, St Peter SD (2018). Outcomes using cryoablation for postoperative pain control in children following minimally invasive pectus excavatum repair. J Laparoendosc Adv Surg Tech A.

[13] Gourlay GK, Wilson PR, Glynn CJ (1982). Pharmacodynamics and pharmacokinetics of methadone during the perioperative period. Anesthesiology.

[14] Kharasch ED (2011). Intraoperative methadone: rediscovery, reappraisal, and reinvigoration?. Anesth Analg.

[15] Morley JS, Bridson J, Nash TP, Miles JB, White S, Makin MK (2003). Low-dose methadone has an analgesic effect in neuropathic pain: a double-blind randomized controlled crossover trial. Palliat Med.

[16] Tajerian M, Leu D, Yang P, Huang TT, Kingery WS, Clark JD (2015). Differential Efficacy of ketamine in the acute versus chronic stages of complex regional pain syndrome in mice. Anesthesiology.

[17] Dolgun H, Turkoglu E, Kertmen H, Gurer B, Yilmaz ER, Comoglu SS, Sekerci Z (2014). Gabapentin versus pregabalin in relieving early post-surgical neuropathic pain in patients after lumbar disc herniation surgery: a prospective clinical trial. Neurol Res.

[18] Bayman EO, Brennan TJ (2014). Incidence and severity of chronic pain at 3 and 6 months after thoracotomy: meta-analysis. J Pain.

[19] Helander EM, Webb MP, Bias M, Whang EE, Kaye AD, Urman RD (2017). A comparison of multimodal analgesic approaches in institutional enhanced recovery after surgery protocols for colorectal surgery: pharmacological agents. J Laparoendosc Adv Surg Tech A.

[20] Ghanem CI, Pérez MJ, Manautou JE, Mottino AD (2016). Acetaminophen from liver to brain: new insights into drug pharmacological action and toxicity. Pharmacol Res.

[21] Vane JR (1971). Inhibition of prostaglandin synthesis as a mechanism of action for aspirin-like drugs. Nat New Biol.

